# Remote diagnosis of surgical-site infection using a mobile digital intervention: a randomised controlled trial in emergency surgery patients

**DOI:** 10.1038/s41746-021-00526-0

**Published:** 2021-11-18

**Authors:** Kenneth A. McLean, Katie E. Mountain, Catherine A. Shaw, Thomas M. Drake, Riinu Pius, Stephen R. Knight, Cameron J. Fairfield, Alessandro Sgrò, Matt Bouamrane, William A. Cambridge, Mathew Lyons, Aya Riad, Richard J. E. Skipworth, Stephen J. Wigmore, Mark A. Potter, Ewen M. Harrison, TWIST Collaborators, K. Baweja, W. A. Cambridge, V. Chauhan, K. Czyzykowska, M. Edirisooriya, A. Forsyth, B. Fox, J. Fretwell, C. Gent, A. Gherman, L. Green, J. Grewar, S. Heelan, D. Henshall, C. Iiuoma, S. Jayasangaran, C. Johnston, E. Kennedy, D. Kremel, J. Kung, J. Kwong, C. Leavy, J. Liu, S. Mackay, A. MacNamara, S. Mowitt, E. Musenga, N. Ng, Z. H. Ng, S. O’Neill, M. Ramage, J. Reed, A. Riad, C. Scott, V. Sehgal, A. Sgrò, L. Steven, B. Stutchfield, S. Tominey, W. Wilson, M. Wojtowicz, J. Yang

**Affiliations:** 1 Department of Clinical Surgery, University of Edinburgh, 51 Little France Crescent, Edinburgh, UK; 2 Centre for Medical Informatics (CMI), Usher Institute, University of Edinburgh, 9 Little France Rd, Edinburgh, UK; 3 Department of Colorectal Surgery, Western General Hospital, Edinburgh, UK; 4 Edinburgh Medical School, University of Edinburgh, Edinburgh, UK

**Keywords:** Diagnosis, Outcomes research, Infectious diseases

## Abstract

Surgical site infections (SSI) cause substantial morbidity and pose a burden to acute healthcare services after surgery. We aimed to investigate whether a smartphone-delivered wound assessment tool can expedite diagnosis and treatment of SSI after emergency abdominal surgery. This single-blinded randomised control trial (NCT02704897) enroled adult emergency abdominal surgery patients in two tertiary care hospitals. Patients were randomised (1:1) to routine postoperative care or additional access to a smartphone-delivered wound assessment tool for 30-days postoperatively. Patient-reported SSI symptoms and wound photographs were requested on postoperative days 3, 7, and 15. The primary outcome was time-to-diagnosis of SSI (Centers for Disease Control definition). 492 patients were randomised (smartphone intervention: 223; routine care: 269). There was no significant difference in the 30-day SSI rate between trial arms: 21 (9.4%) in smartphone vs 20 (7.4%, *p* = 0.513) in routine care. Among the smartphone group, 32.3% (*n* = 72) did not utilise the tool. There was no significant difference in time-to-diagnosis of SSI for patients receiving the intervention (−2.5 days, 95% CI: −6.6−1.6, *p* = 0.225). However, patients in the smartphone group had 3.7-times higher odds of diagnosis within 7 postoperative days (95% CI: 1.02−13.51, *p* = 0.043). The smartphone group had significantly reduced community care attendance (OR: 0.57, 95% CI: 0.34−0.94, *p* = 0.030), similar hospital attendance (OR: 0.76, 95% CI: 0.28−1.96, p = 0.577), and significantly better experiences in accessing care (OR: 2.02, 95% CI: 1.17−3.53, *p* = 0.013). Smartphone-delivered wound follow-up is feasible following emergency abdominal surgery. This can facilitate triage to the appropriate level of assessment required, allowing earlier postoperative diagnosis of SSI.

## Introduction

Surgical site infection (SSI) is one of the most common complications following gastrointestinal surgery^1^, and increasingly occur after discharge with the move towards earlier patient discharge^2^. Early detection, diagnosis, and treatment of SSIs provide the best opportunities to minimise the associated burden of disease and promote rationalised antibiotic use. However, there are substantial clinical challenges due to the requirement for in-person assessment and the subjective nature of diagnostic criteria^3^.

Over three-quarters (78%) of UK adults now own smartphones^4^, expanding the potential for digital health interventions. Given the high frequency of post-operative wound complications, this has become a research focus in telemedicine^5–7^. To date, no clinical trial has been completed to demonstrate the effectiveness or efficacy of digital health interventions used for the purposes of remote wound assessment to identify SSI, nor their implications for patients or the health service. Since the unexpected onset of the COVID pandemic, the routine use of teleconsultations has now become a necessary and accepted practice^8,9^. Remote wound monitoring poses an immense opportunity to understand and improve postoperative community care and minimise the burden of disease for both patients and healthcare services.

This trial aimed to investigate whether a smartphone-delivered wound assessment tool results in earlier diagnosis and treatment of SSI after emergency abdominal surgery. Secondary aims included the evaluation of the impact of this intervention on healthcare services and patient experience of postoperative care.

## Results

### Study population

**Fig. 1:**
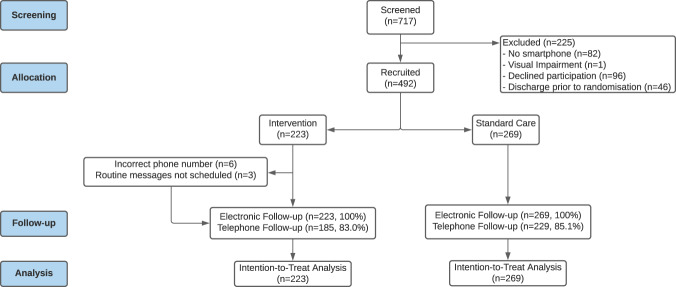
CONSORT flow diagram. The patient flow for the TWIST trial.

Patients were recruited between 26 July 2016 and 4 March 2020, and completion of recruitment preceded any known cases of SARS-CoV-2 infection in the local region. There were 717 patients undergoing emergency abdominal surgery screened for eligibility during this time (Fig. 1). Of those approached, 11.4% (*n* = 82) were excluded due to lacking a smartphone, 13.4% (*n* = 96) declined to participate, and 6.4% (*n* = 46) were discharged prior to randomisation. Of patients excluded due to lack of a smartphone, these were significantly older (76 years, IQR: 66–84) than other patients screened who did have a smartphone (47 years, IQR: 32.8–61, *p* < 0.001).

There were 492 patients recruited to the trial, the majority of whom underwent major surgical procedures (*n* = 414, 84.1%), had a laparoscopic approach (*n* = 361, 73.4%), and had no uncontrolled operative contamination (*n* = 374, 76.0%). These patients were randomly assigned to receive either the smartphone intervention (*n* = 223) or routine postoperative care (*n* = 269). Characteristics showed broadly equal distribution between arms (Table 1).

**Table 1:** Demographic and clinical characteristics of trial participants.

		Routine Care (*n* = 269)	Smartphone (*n* = 223)	Total
Age (years)	Mean (SD)	46.7 (17.1)	41.8 (17.2)	44.5 (17.3)
Sex	Female	149 (55.4)	117 (52.5)	266 (54.1)
	Male	120 (44.6)	106 (47.5)	226 (45.9)
Ethnicity	White	262 (97.4)	213 (95.5)	475 (96.5)
	BAME	7 (2.6)	10 (4.5)	17 (3.5)
Diabetes mellitus	No	256 (95.2)	213 (95.5)	469 (95.3)
	Yes	13 (4.8)	10 (4.5)	23 (4.7)
Body mass index	Not obese	191 (71.0)	161 (72.2)	352 (71.5)
	Obese	78 (29.0)	59 (26.5)	137 (27.8)
	Missing	0 (0.0)	3 (1.3)	3 (0.6)
Immunosuppression	No	257 (95.5)	214 (96.0)	471 (95.7)
	Yes	12 (4.5)	9 (4.0)	21 (4.3)
Operative complexity	Minor or intermediate	41 (15.2)	37 (16.6)	78 (15.9)
	Major or complex major	228 (84.8)	186 (83.4)	414 (84.1)
Operative approach	Laparoscopic	193 (71.7)	168 (75.3)	361 (73.4)
	Open	76 (28.3)	55 (24.7)	131 (26.6)
Operative contamination	Clean-contaminated	204 (75.8)	170 (76.2)	374 (76.0)
	Contaminated/dirty	65 (24.2)	53 (23.8)	118 (24.0)

### Comparison of the smartphone intervention to routine care

**Fig. 2:**
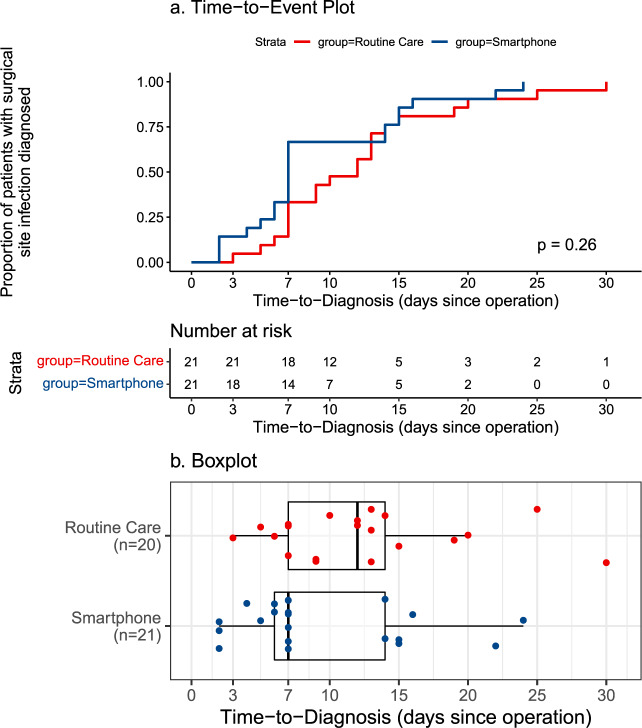
Time-to-diagnosis (days) of surgical-site infection (SSI). The time-to-diagnosis (days) of patients who were diagnosed with SSI within 30-days as (**a**) time-to-event plot (**b**) boxplot.

Overall, 8.3% (*n* = 41) of the cohort developed surgical-site infections (SSI) in the 30-day postoperative period, with no significant difference in the SSI rate between trial arms: 21 (9.4%) in smartphone and 20 (7.4%) in routine care (OR = 1.29, 95% CI: 0.68−2.45, *p* = 0.513) (Table 2). The mean time-to-diagnosis was 9.3 days (SD = 6.3) in the smartphone group, and 11.8 days (SD = 6.7) in the routine care group, which did not demonstrate a significant difference for the primary outcome (−2.5 days, 95% CI: −6.6–1.6, *p* = 0.225). Similarly, there was no overall significant difference in the time-to-diagnosis between trial arms (*p* = 0.340) (Fig. 2a).

**Table 2:** Time-period of diagnosis of surgical-site infection in the trial arms.

	Smartphone (*n* = 223)	Routine Care (*n* = 269)	Odds ratio	*p*
7-Day SSI rate	14 (6.3%)	7 (2.6%)	3.71 (1.02–13.51)	0.043
30-Day SSI rate	21 (9.4%)	20 (7.4%)	1.29 (0.68−2.45)	0.513

On visualisation (Fig. 2b), a bimodal distribution in the smartphone group was observed with grouping around the 7 and 15 days (coinciding with scheduled requests for routine completion). Furthermore, no concerns were identified on routine responses at 3 and 7 days in patients with a later SSI diagnosis in the smartphone arm. A post-hoc analysis was conducted and patients in the smartphone group were found to have a significantly higher odds of diagnosis in first 7 postoperative days (OR: 3.71, 95% CI: 1.02– 13.51, *p* = 0.043) (Table 2).

Healthcare service usage in those who received the smartphone intervention or routine care was compared. Overall, 32 (14.3%) in the smartphone group attended healthcare services regarding their wound (*n* = 25 [11.2%] community services; 7 [3.1%] hospital services), compared to 60 (22.3%) in the routine care group who attended healthcare services regarding their wound (49 [18.2%] community services; 11 [4.1%] hospital services). Patients in the smartphone group had a significantly lower rate of attendance at community care (OR: 0.57, 95% CI: 0.34–0.94, *p* = 0.030), but did not have significantly different rates of attendance at hospital emergency services (OR: 0.76, 95% CI: 0.28–1.96, *p* = 0.577).

Of the 41 surgical-site infections recorded, the majority (78.0%, *n* = 32) were superficial, with 5 deep (12.2%) and 4 (9.8%) organ-space infections. There was no significant difference observed in the smartphone arm in the rate of deep/organ-space infections (OR: 1.25, 95% CI: 0.28–5.53, *p* = 0.769) or major postoperative complications (OR: 0.95, 95% CI: 0.12–7.46, *p* = 0.959) compared to routine care (Supplementary Table 2).

**Fig. 3:**
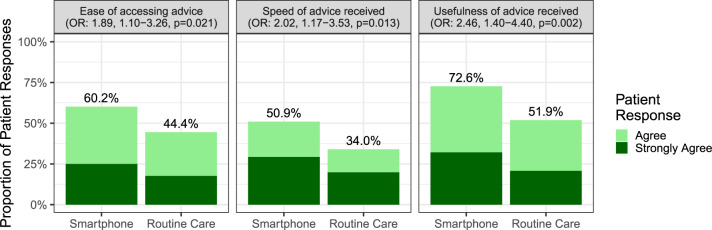
Comparison of patient experience of postoperative care between trial arms. Three measures of patient experience compared between trial arms with regards to the (**a**) ease of access of advice, (**b**) speed of access of advice, and (**c**) usefulness of advice. This displaysthe percentage (%) of patients who reported positive responses to these measures (“agree” or “strongly agree”), and provides an effect estimate (odds ratio [OR]) of the odds of positive response in patients receiving the smartphone intervention, compared to routine care.

Furthermore, when the 30-day patient experience was compared, those in the smartphone arm reported a significantly more positive experience on all measures assessed (Fig. 3). This included access to care (with regards to waiting times [OR: 2.02, 95% CI: 1.17–3.53, *p* = 0.013] and ease of access to advice [OR: 1.89, 95% CI: 1.10–3.26, *p* = 0.021]), as well as the quality of advice received (OR: 2.46, 95% CI: 1.40–4.40, *p* = 0.002).

### Evaluation of the smartphone intervention

Each patient allocated to the smartphone arm automatically received a prompt to routinely complete a survey at days 3, 7, and 15 (or could also additionally complete this at any point within the 30-day period if they had wound concerns). Responses out with the response window for routine requests (wound concerns) represented 10.5% (*n* = 38) of responses, with 52.6% (*n* = 20/38) responses occurring prior to day 3 (median: 2, IQR: 2–4) (Supplementary Fig. 1).

**Fig. 4:**
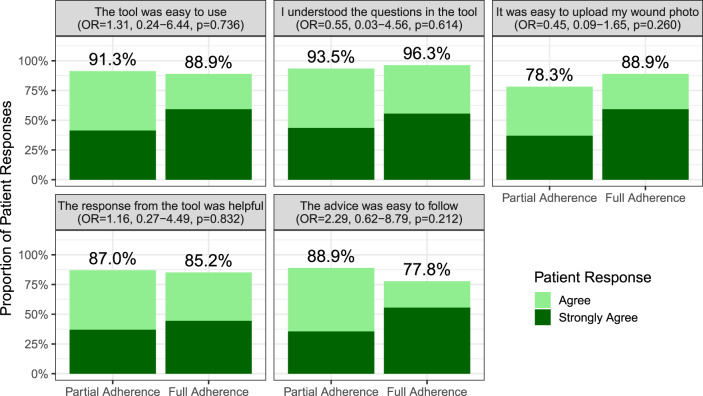
Patient experience of those who used the smartphone tool. The five measures of patient experience in patients who used the intervention (**a**) ease of tool use, (**b**) understandability of tool, (**c**) ease of image upload, (**d**) helpfulness of advice, and (**e**) Feasibility of advice. This has been stratified by adherence: either full adherence (completion of all three routine response requests in the follow-up period) or partial use (any usage of the tool that did not meet the criteria of full adherence). These plots display the percentage (%) of patients who reported positive responses to these measures (“agree” or “strongly agree”) and provides an effect estimate (odds ratio [OR]) of the odds of positive response in patients who displayed full adherence, compared to partial adherence.

Among those randomised to the smartphone arm, 26.0% (*n* = 58) fully adhered to all routine requests, 41.7% (*n* = 93) displayed partial adherence, and finally 32.3% (*n* = 72) did not utilise the tool. There were no significant differences in patient characteristics observed between those who did or did not use the tool (Supplementary Table 3). Furthermore, among patients who used the tool there was a consistently positive experience reported, with no significant differences observed between those who had partial or full adherence (Fig. 4).

Within the smartphone group, there were 21 (9.4%) patients diagnosed with SSI. Of these patients, 7 (33.3%) had used the tool in 48 h prior to diagnosis (5 diagnosed on the same day). All these infections were identified based on patient-reported symptoms (Table 3), categorised as either possible infection (*n* = 4) or probable infection (*n* = 3). Incorporation of wound images offered a significant improvement to specificity from 84.4% (95% CI: 80.5–88.3%) to 93.6% (95% CI: 90.9–96.2%) (Table 3).

**Table 3:** Diagnostic accuracy of SSI diagnosis within 48 h of submission of response, according to the overall evaluation, and the respective patient-reported symptoms and wound image evaluations on clinical review.

		Clinical diagnosis of SSI within 48 h		Diagnostic accuracy (%)				
		Yes	No	AUC (95% CI)	Sensitivity (95% CI)	Specificity (95% CI)	PPV (95% CI)	NPV (95% CI)
Overall Impression	Moderate-high risk	4 (16.0)	21 (84.0)	75.4 (55.5–95.2)	57.1 (20.5–93.8)	93.6 (90.9–96.2)	16.0 (1.6–30.4)	99.0 (97.9–100)
	Low-risk	3 (1.0)	307 (99.0)					
Wound Image	Moderate-high risk	4 (23.5)	13 (76.5)	76.2 (56.3–96.0)	57.1 (20.5–93.8)	95.2 (92.7–97.8)	23.5 (3.4–43.7)	98.9 (97.6–100)
	Low-risk	3 (1.1)	259 (98.9)					
Patient-reported symptoms	Moderate-high risk	7 (12.1)	51 (87.9)	92.2 (90.2–94.2)	100 (100–100)	84.4 (80.5–88.3)	12.1 (3.7–20.5)	100 (100–100)
	Low-risk	0 (0.0)	276 (100.0)					

There was a high rate of concordance (85.6%, *n* = 238/278) between assessment of patient-reported symptoms and wound images. However, evaluations based on wound images were sevenfold less likely to recommend in-patient assessment (1.8% [*n* = 5] vs 12.6% [*n* = 35], McNemar’s OR: 7.00, 95% CI: 2.73–22.89, *p* < 0.001). Overall, 7.5% (*n* = 25/335) of responses from the cohort were identified as requiring assessment due to clinical suspicion of SSI (Table 3). Of these patients, 1 in 6 (*n* = 4/25) was diagnosed with an SSI compared to 1 in 100 (*n* = 3/310) diagnosed with an SSI in those where there were overall no concerning features identified (Table 3).

## Discussion

This trial has demonstrated that a remote wound follow-up digital intervention increased the likelihood of a surgical-site infection being diagnosed within the early postoperative period fourfold, although it did not reduce the absolute time-to-diagnosis of SSI. We also found this was superior to routine care for patients in improving access and perceived quality of care post-discharge, while reducing the rate of community care attendance. In particular, the tool demonstrated high negative predictive discrimination, meaning SSI could be ruled-out with confidence. This demonstrates the intervention was not only effective, but feasible and safe to deliver remote postoperative wound care in patients undergoing emergency abdominal surgery.

Early recognition and treatment of SSI is essential in limiting the progression and overall burden of disease, with evidence that delays to intervention are associated with significantly higher morbidity and mortality^10,11^. Whilst our study was underpowered with regards to its primary outcome (Fig. 2), there remained a significantly higher likelihood of diagnosis in the early postoperative period (within 7 days of surgery) in the intervention group. SSIs represent one of the most common healthcare-acquired infections with the majority identified post-discharge^2^. Diagnosis and treatment in the early postoperative period would be expected to yield substantial benefits at a population-level^12^. Nonetheless, there was still a significant minority (one third) of those in the intervention arm who were diagnosed with SSI later in the postoperative period (after 7 postoperative days). There was no evidence of infection observed in previous responses for these patients, which aligns with evidence that late-onset SSIs represent a distinct pathophysiological process^13^. This cannot exclude the possibility of gradual progression of a sub-clinical infection or insufficient sensitivity or frequency of responses related to the tool itself. Further trials to reduce time-to-diagnosis of SSI should consider this potential bimodal distribution in the incidence of SSI in the early postoperative period.

There is substantial interest in the use of patient-reported symptoms^14^ and wound images^5,7^ for the purposes of remote diagnosis of SSI, although their relative value in the clinical evaluation was previously unclear. The high specificity combined with the low rate of false negatives in the triage of wounds for further assessment of SSI in this trial compares favourably to those expected in similar screening tests for mammography^15^ or pulmonary embolism^16^. Despite being designed for high sensitivity, the use of patient-reported symptoms alone successfully classified 82.6% (*n* = 276/334) of responses as not requiring clinical assessment, with no false negatives. However, as patient-reported symptoms are known to have high sensitivity for SSI^14^, there remains the potential iatrogenic harm due to overdiagnosis of infections that would otherwise self-resolve. A large-scale, multicentre trial would be required to detect differences in the severity or Clavien-Dindo grade associated with SSI, however, there were no significant differences were observed on these measures (Supplementary Table 2). Together with an overall SSI rate consistent with previous national estimates^17^, this does provide evidence suggesting that there was no clear Hawthorne effect^12^ or measurement bias leading to an overdiagnosis of clinically insignificant surgical-site infections within TWIST.

The incorporation of wound images to the assessment process offered a clinically significant benefit to the specificity and demonstrated a high rate of concordance (85.6%) with patient-reported symptoms. While just 1% (*n* = 3/307) of wound images were rated as “no concerns” despite a clinical diagnosis of SSI within the following 48 h, this nonetheless represented 42.9% (*n* = 3/7) of all infections. Whilst this remains consistent with previous literature exploring image-based diagnosis of SSI^6,18,19^, it is recognised that the diagnostic criteria for SSI remain subjective, with inter-rater disagreement observed between “gold-standard” in-person assessments^20,21^. Furthermore, it should also be recognised that particularly organ-space SSI may not have any associated visual evidence, further complicating their remote diagnosis. There is currently sparce evidence regarding the minimally acceptable criteria for patients and healthcare teams regarding the diagnostic accuracy of digital health interventions, with few implemented in routine clinical practice.

TWIST is a pragmatic clinical trial providing evidence of the effectiveness of this digital health intervention on postoperative care and incorporates assessment of the wider implications on health service delivery. Emergency abdominal surgery represents an initial use-case given the higher rates of SSI, but there are broader applications in areas of healthcare, particularly as patients increasingly expect to be involved and empowered regarding their healthcare^22^. This intervention adopts a patient-driven approach to wound assessment, empowering individuals in their own postoperative recovery, as well as enhancing communication with surgical teams in the early postoperative phase. Finally, the digital nature of this intervention provides further opportunities for automating the wound assessment procedure, and in particular using machine learning computer vision approaches for image assessment^7^.

There were several limitations to this clinical trial. Firstly, whilst the randomisation process generated balanced groups in terms of patient characteristics (Table 1), there remained a small but notable deviation from the intended 1:1 allocation (45:55). This was identified following the completion of the trial, and was due to natural variation in the simple computer-generated randomisation sequence. While there is no evidence that this affected the validity of results, it reduced the power to detect a significant difference in the primary outcome. Secondly, one third of patients (*n* = 72/223) randomised to the smartphone tool did not submit information regarding their wounds over the 30-day postoperative period. However, there was no evidence of significant bias in the patient or operative characteristics between those who were compliant with the intervention and those who were not (Supplementary Table 3). Therefore, while significant benefits were observed in the intervention arm, these results represent an underestimation of the optimal efficacy but a pragmatic understanding of the effectiveness in practice. Thirdly, only one senior clinician (EMH) reviewed responses to provide clinical recommendations, and so given the subjective nature of SSI, it is possible that different clinical staff may provide different recommendations^19^. Further work is required to investigate inter-rater reliability among those who would use this system in practice, for example, different professions (medical or nursing staff) and stages of training. Finally, the schedule for routine completion was chosen to encompass the peak incidence of infection and to minimise volunteer bias. However, the grouping of SSI diagnosis around the scheduled day 7 and 15 responses may indicate increasing the frequency of routine responses would enhance the effectiveness of the intervention in reducing time-to-diagnosis of SSI. This must be balanced with the burden imposed to patients and the service to review in a timely manner.

The COVID-19 pandemic has imposed an enormous disruption to the delivery of surgical care, with the routine use of teleconsultations becoming a necessity^9^. This has accelerated what may have otherwise taken decades of integrating telemedicine into routine clinical practice, with high rates of satisfaction from patients and healthcare staff^8,9^. Policy makers and healthcare planners should anticipate an ongoing and increasing demand for these services following the pandemic. This trial has demonstrated that remote postoperative surveillance, can be safely delivered while reducing health service usage. This and similar digital health interventions for remote postoperative monitoring may further improve Enhanced Recovery after Surgery (ERAS) programmes by increasing safety and confidence in early discharge^23^, and improve SSI surveillance efforts^24,25^. With increasing burdens placed upon community and hospital services from other sources, minimising unnecessary attendances and early intervention in the SSI disease process can provide substantial benefits for healthcare systems and patients themselves. This will be of increased importance as healthcare systems contend with the ongoing, indirect and secondary effects of the COVID-19 pandemic^26^.

Furthermore, remote healthcare has immense potential to improve both access and perceived quality of care, as observed in this trial (Fig. 3) and other studies^27,28^. This may have the greatest value in rural or underserved communities, where barriers already exist in access to in-person care^29,30^. However, we must ensure that a digital divide does not perpetuate existing inequities in care. Disadvantaged groups who potentially stand to benefit most from improved access to care from telehealth (such as those who are elderly^31^, or from low socioeconomic status^32^ or minority ethnic backgrounds^33^) may lack sufficient digital access and/or literacy to gain equitably from these interventions. Notably, 11% of patients in TWIST were ineligible due to a lack of a smartphone, and these patients were disproportionately older. As such, given the mean age of patients within TWIST was 44.5 years old, care should be taken if generalising these results to older emergency surgical patients. Patient-public involvement from across societal groups will be essential in the development and further implementation of digital health interventions in practice. This should incorporate best practice identified from service changes already being piloted out of necessity during the ongoing COVID-19 pandemic^34^. Further work is underway to understand how to optimise implementation of digital postoperative surveillance into routine practice, including promoting proactive engagement from all patients.

Remote postoperative care has become commonplace internationally since the advent of the COVID-19 pandemic, with many health systems already having implemented this within their services. Focus now needs to be on how these interventions can be evidenced and evaluated^35^, and sustainably maintained moving forward. The TWIST trial provides a comprehensive evaluation of smartphone-delivered wound assessment for surgical-site infection and has demonstrated that patient-driven digital postoperative wound follow-up can be feasibly delivered. This can facilitate triage of patients to the appropriate level of assessment required, allowing diagnosis of SSI earlier in the postoperative period. Furthermore, patients demonstrate a clear preference and positive opinion of remote wound assessment in postoperative care. As the global community recovers from the COVID-19 pandemic, this presents an ideal circumstance to capitalise on greater familiarity and acceptance of telemedicine among both healthcare staff and patients in order to further improve postoperative care.

## Methods

### Study design and participants

Tracking wound infection with smartphone technology (TWIST) was a 2-arm, parallel design, pragmatic randomised control trial conducted across two tertiary hospitals in a large health board in the United Kingdom (UK), serving a mixed urban and rural population of over 800,000.

Adult inpatients (aged 16 years or older) who underwent emergency abdominal surgery (on the same admission as diagnosis) were screened for eligibility. Key inclusion criteria were smartphone ownership (with internet access) and capacity to provide informed consent. Patients were excluded based on self-reported visual impairment which would prevent interaction with online resources. Written consent for each patient was obtained in line with Good Clinical Practice (GCP) standards.

TWIST was reviewed and approved by South-East Scotland Research Ethics Committee (Number: 16/SS/0072). The trial was pre-registered on ClinicalTrials.gov (NCT02704897, registration: 10/03/16), and the protocol published^36^. This study is reported according to the Consolidated Standards of Reporting Trials (CONSORT) guideline^37^. A pre-planned internal pilot study in the first 80 patients recruited was conducted to ensure the trial design was practical and deliverable.

The trial was funded by the University of Edinburgh and conducted with support from existing staff and resources in the NHS Lothian health board. All authors had full access to all the data in the study and were involved in data interpretation and writing of the report. The corresponding author had final responsibility for the decision to submit for publication

### Randomisation and masking

Eligible participants who provided informed consent were randomly allocated (1:1 ratio) to receive either routine postoperative care or the addition of a smartphone-delivered wound assessment tool. The random number sequence was computer-generated and integrated into the data collection platform. No stratification or minimisation was used. Research team members performing randomisation did not have access to the sequence, and the allocation process was automated.

Due to the nature of the intervention, patients and healthcare practitioners with which the patient had contact following discharge were not blinded to the allocation status. However, clinical teams and outcome assessors at 30-days were blinded to allocation status.

### Procedures

Enroled patient details (including mobile telephone number) were entered into a secure Research Electronic Data Capture (REDCap) database. Furthermore, additional sociodemographic and operative data were collected based on clinically relevant risk factors for SSI. These included age, sex, ethnicity (White, or Black, Asian and other minority ethnic groups [BAME]), obesity (body mass index [BMI] ≥ 30 kg/m^2^), diabetes mellitus, immunosuppression (known HIV positive status, corticosteroids, chemotherapy received within 6 weeks, or other immunomodulating drugs), operative approach (open or laparoscopic), operative complexity (minor/intermediate or major/complex major according to the BUPA Schedule of Procedures^38^), and CDC surgical wound classification^3^ (clean/clean-contaminated, or contaminated/dirty).

**Fig. 5:**
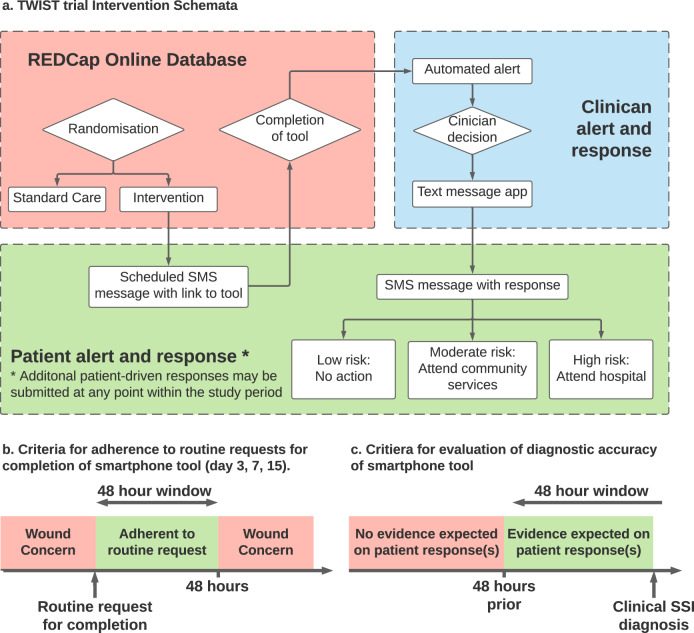
TWIST Trial definitions and processes. **a** The intervention schemata which demonstrates the process of the digital health intervention. This outlines how the clinical team was alerted whenever a patient response was submitted (whether patient-initiated or a routine request on days 3, 7, of 15), and the communication to patients when a clinical decision on the risk of surgical-site infection was made. This also depicts the criteria for assessing secondary outcomes in the smartphone arm: **b** how adherence to routine requests for completion of smartphone tool was determined, and **c** how diagnostic accuracy of clinical assessments of responses submitted using the smartphone tool was determined.

Patients who were randomised to the routine care arm received no further communication from the research team prior to 30-day follow-up. Patients randomised to the smartphone group had a personal hyperlink automatically sent by short-messaging system (SMS) to their smartphones. This allowed immediate access to a secure online wound assessment tool for the 30-day postoperative period (starting from postoperative day 1), to facilitate patient-driven contact regarding any wound concerns (Fig. 5A). Furthermore, patients were scheduled to receive prompts to complete the tool at postoperative days 3, 7, and 15 irrespective of wound concerns. Patients were asked to complete these routine requests whether or not they were an inpatient at the time.

The smartphone-delivered wound assessment tool required submission of (1) SSI-specific patient-reported symptoms and (2) images of their wound (Supplementary Table 1)^36^. Patient-reported symptoms were based on the Centers for Disease Control and Prevention (CDC) classification criteria, and the ASEPSIS model (Additional treatment, Serous discharge, Erythema, Purulent exudate, and Separation of the deep tissues, the Isolation of bacteria, and the duration of Inpatient Stay)^3,39^. This addressed evidence of SSI and/or resultant systemic infection that could be considered apparent to the patient, while minimising the burden of completion. Participants were asked to upload at least one contemporaneous photograph of their wound for each use of the wound assessment tool.

Submission of a response by a patient generated an automated alert for review of the information. Automated classification of patient-reported symptoms was performed with a prespecified clinical algorithm^36^ Given the investigative nature of this study and patient safety as a priority, a senior clinician (EMH) reviewed all patient-reported symptom responses, wound images, and algorithm outputs in real time. The evidence of SSI on patient-reported symptoms and wound images was classified separately as either: low-risk (no apparent evidence of SSI), medium-risk (possible evidence of SSI), or high-risk (probable evidence of SSI). Clinical advice was based on patient-reported symptoms, with wound images providing supplementary information, providing three possible classifications: (1) that there was no clear evidence of SSI present, but to attend healthcare services or submit a further form if ongoing concerns (overall low-risk); (2) to attend community healthcare services for clinical review (overall medium-risk); or (3) to attend emergency services at their treatment centre for clinical review (overall high-risk). These recommendations were agreed in collaboration with the emergency surgical team. Submission of this clinical recommendation was performed within a target of 24 h from the time of first alert, with this response communicated to the patient through SMS on an automated basis on submission by the reviewing clinician (Fig. 5A). Wound image classification with convolutional neural networks was performed in an embedded study. Outputs from this were not available for real-time patient assessment, and these will be reported separately.

### Outcomes and definitions

The primary outcome measure was time-to-diagnosis (days) of the SSI (superficial, deep or organ-space)^3^ within the 30-day postoperative period. Secondary outcomes considered healthcare attendance for wound review, and Clavien-Dindo grade of SSI-associated complications^40^ (divided into “minor” [Grade I-II] and “major” [Grade III-V]) and patient experience at 30-day follow-up (delivered via a separate questionnaire alongside the 30-day follow-up^36^).

All patients enroled were assessed by clinicians blinded to the randomisation status through three independent approaches. Firstly, patients received 30-day postoperative follow-up following a standardised format. This reviewed the occurrence of patient-reported symptoms related to their wound over the 30-day period, any healthcare attendances and any treatments received in that time. Secondly, all participants were provided a log on enrolment where any wound reviews conducted in the community could be recorded and returned to the trial team in a pre-paid envelope. This included the date of assessment, whether an SSI was diagnosed, and any therapeutic intervention performed or commenced. Finally, a data-enabled trial approach utilised the electronic patient record for each patient to identify healthcare attendances and any diagnosis of SSI (including all microbiology results from swabs taken in the community or hospital). On the basis of these three sources of information, two independent, blinded clinical researchers (trained in applying the CDC criteria^3^) determined if an SSI was present. The clinician involved in the assessment of patient responses (EMH) was not involved in this decision process, and these blinded outcome assessors did not have access to any patient responses or associated clinical recommendations from the smartphone arm.

For those allocated to the smartphone arm, further secondary outcomes regarding the wound assessment tool were evaluated based on patient adherence and diagnostic accuracy. Responses submitted within 48 h following a routine request (postoperative days 3, 7, and 15) were considered as adherent responses (Fig. 5B). All other responses out with these windows were considered as a submission of a wound concern. Full adherence was defined as the completion of all three routine response requests in the follow-up period. In comparison, partial adherence was defined as any usage of the tool that did not meet the criteria of full adherence, and non-adherence as non-completion of any responses.

For determining the sensitivity and specificity of the intervention, a pragmatic approach was taken. Responses submitted in the 48 h prior to a clinical diagnosis of surgical-site infection were expected to have evidence of SSI that would be identifiable on submitted responses (Fig. 5C). As such, any response evaluated as “no concerns” with an SSI clinically diagnosed in the following 48 h was considered a false negative result. Similarly, any response evaluated as “possible” or “probable” SSI yet not leading to a subsequent clinical diagnosis within 48 h was considered a false-positive result.

### Statistical analysis

Analyses were conducted according to a pre-specified analysis plan, on an intention-to-treat basis (unless identified as post hoc). We calculated that a sample size of 490 patients were required to demonstrate the superiority of the smartphone intervention (1-day difference in time-to-diagnosis), with a power of 90% and significance level of 5%. This assumed a 10% SSI rate in both groups (in line with national data^17^), a standard deviation of 1 day, and an attrition rate of 10%. Interim results were viewed following completion of the internal pilot study, however, no statistical testing was performed and so no adjustments were made for interim analysis.

Continuous data were summarized as mean (standard deviation) or median (interquartile range) based on visual and statistical evaluation for normality, with appropriate parametric or non-parametric tests performed. The primary outcome (time-to-diagnosis) was compared using a two-sample *t*-test, as well as a Cox proportional hazard regression analysis as a secondary outcome. Categorical data were cross-tabulated, and differences tested using Pearson’s chi-squared test, Fisher’s exact test, or McNemar’s chi-squared test as appropriate. Patient experience was dichotomised into positive and non-positive (neutral or negative) responses to questions.

Multivariable logistic regression models were constructed for evaluating the diagnostic accuracy of clinical diagnosis of SSI within 48 h based on clinical evaluation of patient-reported symptoms, wound images, and the overall impression of a submitted response. Data are censored at the point of clinical SSI diagnosis, and results are presented using the area under the curve (AUC), sensitivity, specificity, positive predictive value (PPV), and negative predictive value (NPV).

Statistical significance was set a priori at *p* < 0.05, and all statistical analyses performed in R version 3.6.1 (R Foundation for Statistical Computing, Vienna, Austria).

### Reporting summary

Further information on research design is available in the Nature Research Reporting Summary linked to this article.

## Supplementary information

Supplementary Material

Reporting Summary

## Data Availability

The datasets generated during and/or analysed during the current study are available from the corresponding author on reasonable request.

## Code availability

The code generated and used during the current study is available from the corresponding author on reasonable request.
