# Supplementary material for: Remote diagnosis of surgical-site infection using a mobile digital intervention: a randomised controlled trial in emergency surgery patients
Source: NPJ Digit Med. 2021 Nov 18;4:160. doi: 10.1038/s41746-021-00526-0 (PMC8602321; doi:10.1038/s41746-021-00526-0)

Supplement:

Supplementary Table 1: Smartphone-delivered wound assessment tool

Question	Response
1. Is the pain worse than immediately after the operation?	No, Yes
2. Is there new redness around your wound site excluding the wound itself?	No, Yes
3. Is there more swelling around your wound site than at the time of surgery?	No, Yes
4. Are you experiencing a new burning sensation or heat at the wound site?	No, Yes
5. Is there liquid coming from the wound site? If so, please select which option best describes the liquid.	No, Yes – clear, Yes – bloody, Yes – yellowish, Yes – thick/yellow, Yes – green/brown
6. Is your wound opening or gaping?	No, Yes
7. Have you experienced fevers in the last 24 hours?	No, Yes
8. Please upload a photograph of your wound.	[Photograph]

Supplementary Table 2: 30-day surgical site infection (SSI) outcomes.

		30-day Surgical Site Infection (SSI)		
		Smartphone (n=21)	Routine Care (n=20)	p
SSI Severity	Superficial	16 (76.2%)	16 (80.0%)	0.555
	Deep	2 (9.5%)	3 (15.0%)	
	Organ-space	3 (14.3%)	1 (5.0%)	
SSI-associated complication rate (Clavien-Dindo)	Grade I-II	19 (90.5%)	18 (90.0%)	0.959
	Grade III-IV *	2 (9.5%)	2 (10.0%)	

* No deaths associated with surgical-site infection were recorded within 30-days.

Supplementary Table 3: Characteristics associated with patient compliance with the intervention.

		Use of the smartphone tool		
		No (n=72)	Yes (n=151)	p
Age (years)	Mean (SD)	43.2 (17.1)	41.1 (17.3)	0.356
Sex	Female	36 (50.0)	81 (53.6)	0.611
	Male	36 (50.0)	70 (46.4)	
Ethnicity	White	70 (97.2)	143 (94.7)	0.395
	BAME	2 (2.8)	8 (5.3)	
Diabetes Mellitus	No	69 (95.8)	144 (95.4)	0.874
	Yes	3 (4.2)	7 (4.6)	
Body Mass Index	Not Obese	51 (71.8)	110 (73.8)	0.755
	Obese	20 (28.2)	39 (26.2)	
Immunosuppression	No	70 (97.2)	144 (95.4)	0.510
	Yes	2 (2.8)	7 (4.6)	
Operative Complexity	Minor or Intermediate	12 (16.7)	25 (16.6)	0.983
	Major or Complex major	60 (83.3)	126 (83.4)	
Operative Approach	Laparoscopic	52 (72.2)	116 (76.8)	0.456
	Open	20 (27.8)	35 (23.2)	
Operative Contamination	Clean-Contaminated	59 (81.9)	111 (73.5)	0.166
	Contaminated / Dirty	13 (18.1)	40 (26.5)	
30-day Surgical Site Infection (SSI)	No	63 (87.5)	139 (92.1)	0.276
	Yes	9 (12.5)	12 (7.9)	

Supplementary Figure 1: Histogram of the timing of submissions of routine responses or wound concerns, according to adherence.

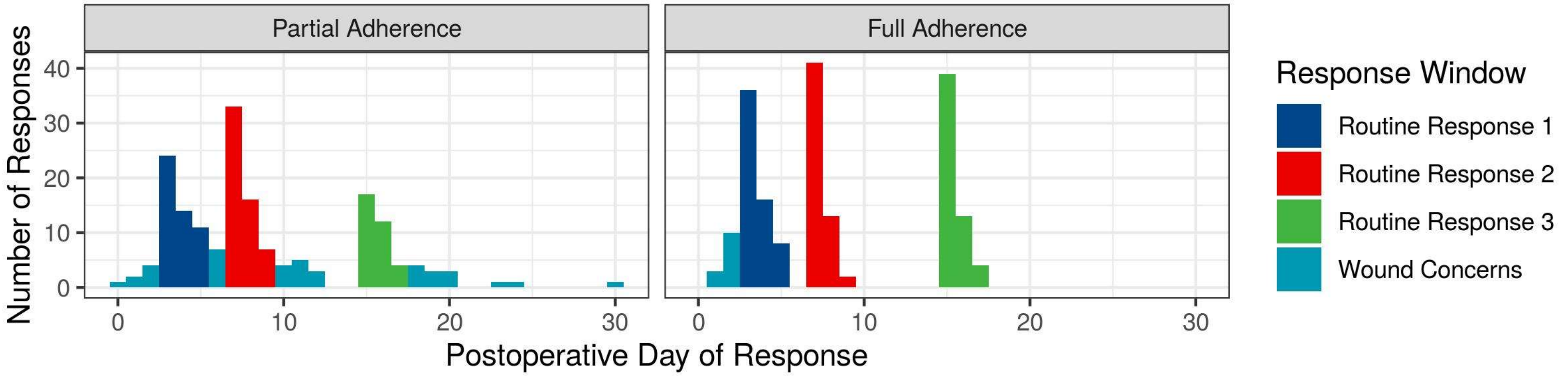

Supplement: Supplementary file 1 — Supplementary Material [file 41746_2021_526_MOESM1_ESM.pdf]
