# Supplementary material for: Remote diagnosis of surgical-site infection using a mobile digital intervention: a randomised controlled trial in emergency surgery patients
Source: NPJ Digit Med. 2021 Nov 18;4:160. doi: 10.1038/s41746-021-00526-0 (PMC8602321; doi:10.1038/s41746-021-00526-0)
Supplement: Supplementary file 2 — Reporting Summary [file 41746_2021_526_MOESM2_ESM.pdf]

nature portfolio

Corresponding author(s): Ewen Harrison

Last updated by author(s): Aug 19, 2021

Reporting Summary

Nature Portfolio wishes to improve the reproducibility of the work that we publish. This form provides structure for consistency and transparency in reporting. For further information on Nature Portfolio policies, see our Editorial Policies and the Editorial Policy Checklist.

Statistics

For all statistical analyses, confirm that the following items are present in the figure legend, table legend, main text, or Methods section.

n/a	Confirmed	
☐	☒	The exact sample size (n) for each experimental group/condition, given as a discrete number and unit of measurement
☐	☒	A statement on whether measurements were taken from distinct samples or whether the same sample was measured repeatedly
☐	☒	The statistical test(s) used AND whether they are one- or two-sided<br>*Only common tests should be described solely by name; describe more complex techniques in the Methods section.*
☐	☒	A description of all covariates tested
☐	☒	A description of any assumptions or corrections, such as tests of normality and adjustment for multiple comparisons
☐	☒	A full description of the statistical parameters including central tendency (e.g. means) or other basic estimates (e.g. regression coefficient) AND variation (e.g. standard deviation) or associated estimates of uncertainty (e.g. confidence intervals)
☐	☒	For null hypothesis testing, the test statistic (e.g. F, t, r) with confidence intervals, effect sizes, degrees of freedom and P value noted<br>*Give P values as exact values whenever suitable.*
☒	☐	For Bayesian analysis, information on the choice of priors and Markov chain Monte Carlo settings
☒	☐	For hierarchical and complex designs, identification of the appropriate level for tests and full reporting of outcomes
☒	☐	Estimates of effect sizes (e.g. Cohen's d, Pearson's r), indicating how they were calculated

Our web collection on statistics for biologists contains articles on many of the points above.

Software and code

Policy information about availability of computer code

Data collection	REDCap (Research Electronic Data Capture) web application
Data analysis	R version 3.6.1 (R Foundation for Statistical Computing, Vienna, Austria)

For manuscripts utilizing custom algorithms or software that are central to the research but not yet described in published literature, software must be made available to editors and reviewers. We strongly encourage code deposition in a community repository (e.g. GitHub). See the Nature Portfolio guidelines for submitting code & software for further information.

Data

Policy information about availability of data

All manuscripts must include a data availability statement. This statement should provide the following information, where applicable:

- Accession codes, unique identifiers, or web links for publicly available datasets
- A description of any restrictions on data availability
- For clinical datasets or third party data, please ensure that the statement adheres to our policy

The datasets generated during and/or analysed during the current study are available from the corresponding author on reasonable request.

Field-specific reporting

Please select the one below that is the best fit for your research. If you are not sure, read the appropriate sections before making your selection.

☒ Life sciences ☐ Behavioural & social sciences ☐ Ecological, evolutionary & environmental sciences

For a reference copy of the document with all sections, see nature.com/documents/nr-reporting-summary-flat.pdf

Life sciences study design

All studies must disclose on these points even when the disclosure is negative.

Sample size	Sample size calculation as described in Methods (page 7, line 24)
Data exclusions	No data were excluded for patients post-randomisation.
Replication	First clinical trial in area - no replication
Randomization	Randomly allocated (1:1 ratio) to receive either routine postoperative care or the addition of a smartphone-delivered wound assessment tool. The random number sequence was computer-generated and integrated into the data collection platform. No stratification or minimisation was used. Research team members performing randomisation did not have access to the sequence, and the allocation process was automated.
Blinding	Due to the nature of the intervention, patients and healthcare practitioners with which the patient had contact following discharge were not blinded to the allocation status. However, clinical teams and outcome assessors at 30-days were blinded to allocation status.

Reporting for specific materials, systems and methods

We require information from authors about some types of materials, experimental systems and methods used in many studies. Here, indicate whether each material, system or method listed is relevant to your study. If you are not sure if a list item applies to your research, read the appropriate section before selecting a response.

Materials & experimental systems

n/a	Involved in the study	
☒	☐	Antibodies
☒	☐	Eukaryotic cell lines
☒	☐	Palaeontology and archaeology
☒	☐	Animals and other organisms
☐	☒	Human research participants
☐	☒	Clinical data
☒	☐	Dual use research of concern

Methods

n/a	Involved in the study	
☒	☐	ChIP-seq
☒	☐	Flow cytometry
☒	☐	MRI-based neuroimaging

Human research participants

Policy information about studies involving human research participants

Population characteristics	There were 492 patients recruited to the trial - mean age = 44.5 (SD: 17.3), 54.1% female (n=266). The majority underwent major surgical procedures (n=414, 84.1%), had a laparoscopic approach (n=361, 73.4%), and had no uncontrolled operative contamination (n=374, 76.0%).
Recruitment	Recruitment was active at two tertiary hospitals in a large health board in the United Kingdom (UK), serving a mixed urban and rural population of over 800,000. Adult inpatients (aged 16 years or older) who underwent emergency abdominal surgery (on the same admission as diagnosis) were screened for eligibility.
Ethics oversight	South-East Scotland Research Ethics Committee (Number: 16/SS/0072)

Note that full information on the approval of the study protocol must also be provided in the manuscript.

Clinical data

Policy information about clinical studies

All manuscripts should comply with the ICMJE guidelines for publication of clinical research and a completed CONSORT checklist must be included with all submissions.

Clinical trial registration	ClinicalTrials.gov (NCT02704897, registration: 10/03/16)

Study protocol	https://bmjopen.bmj.com/content/9/10/e029620
Data collection	Recruitment was active at two tertiary hospitals in a large health board in the United Kingdom (UK), serving a mixed urban and rural population of over 800,000.
Outcomes	The primary outcome measure was time-to-diagnosis (days) of the SSI (superficial, deep or organ-space) within the 30-day postoperative period according to CDC diagnostic criteria. Secondary outcomes considered healthcare attendance for wound review, Clavien-Dindo grade of SSI-associated complications (divided into "minor" [Grade I-II] and "major" [Grade III-V]) and patient experience at 30-day follow-up (delivered via a separate questionnaire alongside the 30-day follow-up).
